# miR-371b-5p promotes cell proliferation, migration and invasion in non-small cell lung cancer via SCAI

**DOI:** 10.1042/BSR20200163

**Published:** 2020-11-13

**Authors:** Xue Luo, Xiaolei Zhang, Jianming Peng, Yan Chen, Wenhui Zhao, Xiuling Jiang, Landi Su, Mingqi Xie, Bo Lin

**Affiliations:** 1 School of Medicine, Yangzhou Polytechnic College, Yangzhou, Jiangsu 225009, China; 2 Department of Basic Medicine, Jiangsu College of Nursing, Huai’an, Jiangsu 223005, China

**Keywords:** biomarker, non-small cell lung cancer, therapeutic target

## Abstract

**Objective:** Multiple gene targets have been reported for treatment of non-small cell lung cancer (NSCLC), however, the accompanying genetic tolerance was reported increasingly. Therefore, it is important to find new biomarkers or therapeutic targets in treatment of NSCLC.

**Methods**: The expression levels of miR-371b-5p were detected by qRT-PCR in NSCLC tissues and cell lines. To evaluate the effect of miR-371b-5p on NSCLC progression, we first transfected the miR-371b-5p inhibitor for construction of the miR-371b-5p down-regulated cell model. Then the cell proliferation, migration, invasion and cell apoptosis were detected. In addition, the expression levels of adhesion factors were detected. The target gene of miR-371b-5p was identified by bioinformatics analysis, and rescue experiment was conducted to validate the effect of miR-371b-5p on proliferation, migration and invasion of NSCLC.

**Results**: Our findings revealed that the miR-371b-5p was overexpressed in NSCLC and could markedly promote the cell proliferation, migration and invasion. Expression levels of both intercellular adhesion molecule 1 (ICAM-1) and vascular cell adhesion molecule-1 (VCAM-1) were significantly down-regulated when treated by miR-371b-5p inhibitor. Moreover, dual-luciferase reporter assay showed that the miR-371b-5p targeted SCAI in regulation of cell proliferation, migration and invasion, and the expression of miR-371b-5p was negatively associated with SCAI in NSCLC tissues and cell lines. Rescue experiment revealed that the miR-371b-5p could rescue the effect of SCAI on cell proliferation, migration and invasion.

**Conclusion**: Our results suggest that the miR-371b-5p and SCAI may serve as novel prognostic biomarkers and therapeutic targets for NSCLC.

## Introduction

Lung cancer is reported to be one of the leading causes of deaths in both males and females worldwide [1]. Among which, the non-small cell lung cancer (NSCLC) represents approximately 85% of all lung cancer types [2]. Despite developments in the clinical oncology technology, the prognosis of NSCLC is poor with only 15% of 5-year survival rate [3]. Increasing studies have proved that the progression of NSCLC is closely associated with a variety of gene abnormalities [4]. Therefore, the identification of diver oncogenic alterations as well as the involved key pathways is of great importance in understanding the lung cancer pathogenesis and development. Although recent study illustrates that several gene targets have been reported for treatment of NSCLC, however, the accompanying genetic tolerance are reported increasingly [5].

MicroRNA (miRNA) is composed of 19-25 nucleotides and is involved in modulation of gene transcription by binding to 3′ untranslated region (3′UTR) of the target genes [5]. It plays an important role in deregulation of malignancies [6–8], which is closely associated with oncogene and progression of tumors through affecting the cellular process, such as cell growth and metastasis [9,10]. Hence, miRNAs are considered as important biomarkers and therapeutic targets in several types of cancers [11,12]. In NSCLC, the miR-199a-5p was reported to be targeted with MAP3K11 to inhibit cell progression [13]. miR-199a-3p can restrain the NSCLC cell proliferation and promote apoptosis by targeting CDK4 [14]. miR-532-3p has been reported to augment the malignance of NSCLC by enhancing HMGA2 expression [15].

Of note, increasing evidence proved that the miR-371b-5p was also associated with the progression of several types of cancers. For example, it can promote the progression of bladder cancer by targeting FUT4 [16]. For currently incurable lung diseases, the alveolar progenitor type II cell (ATIIC)-derived exosome miR-371b-5p serves as a niche signaling during ATIIC proliferation and survival, which promotes re-epithelialization of injured alveoli [17]. Nevertheless, the function of miR-371b-5p in NSCLC remains unknown.

In the present study, we detected the expression levels of miR-371b-5p both in NSCLC tumor tissues and cell lines by using quantitative approach. Then the genetic engineering method was applied to infer the expression of miR-371b-5p in NSCLC cell lines (H1299 and H466). The biological functions of miR-371b-5p in cell proliferation, migration and invasion, as well as its direct target and associated signaling pathways were examined in the present study.

## Materials and methods

### Patients and NSCLC samples

The NSCLC tissues and the adjacent normal lung tissues were collected from 32 patients (16 patients of low miR-371b-5p expression; 16 patients of high miR-371b-5p expression) who underwent surgery between January 2015 and December 2016 in our hospital. All patients did not accept neoadjuvant therapy before surgery. The tumor node metastasis (TNM) was determined by histopathology. The health status of them were checked or followed-up by phone or questionnaire. The last follow-up date was October 2019.

### Cell culture

The normal human bronchial epithelial cells (NHBE) and human NSCLC cell lines (H1299, Pc9, H466, H292) were purchased from Sigma (Maryland, U.S.A.). All these cell lines were cultured in Dulbecco’s modified Eagle’s medium (DMEM, Gibco, New York, U.S.A.) supplemented with 5% fetal bovine serum (FBS, Gibco, New York, U.S.A.) at 37°C and 95% humidity.

### RNA isolation and qRT-PCR

Total RNA was extracted and purified by using the TRIzol kit (Invitrogen, California, U.S.A.) on the basis of the manufacturer’s instructions. Then the level of RNA transcripts was detected by the qRT-PCR analysis. Briefly, the total RNA was converted into cDNA by using the M-MLV reverse transcriptase (Promega, Madison, Wisconsin, U.S.A.). β-actin was used as the internal control. The specific primers in the present study used for amplification were listed in Table 1.

**Table 1:** Clinical parameters associated with miR-371b-5p expression among patients with lung cancer

Characteristics	Number	miR-371b-5p expression		*P*-value
		Low (*n*=16)	High (*n*=16)	
**Sex**				0.485
Male	19	7	12	
Female	13	9	4	
**Age (years)**				0.293
≤60	20	8	12	
>60	12	8	4	
**Tumor size, cm**				0.804
≤4	17	11	6	
>4	15	5	10	
**Pathological staging**				0.035
I + II	5	4	1	
III + IV	27	12	15	
**Mestasis**				0.026
Yes	26	16	15	
No	5	5	1	
**Smoking**				0.553
Yes	12	2	10	
No	20	14	6	

### Western blot analysis

The total proteins from NSCLC samples or cells were extracted first and separated by sodium dodecyl sulfate/polyacrylamide gel electrophoresis (SDS/PAGE). Then, they were transferred into the polyvinylidene fluoride (PVDF) membranes. After blocking by skimmed milk, the membranes were incubated by the primary antibodies listed as follows: anti-Bax (1:500, Arigo Biolaboratories, Shanghai, China), anti-Bcl-2 (1:500, Arigo Biolaboratories, Shanghai, China), anti-Cleaved caspase-3 (1:500, Abcam, Wuhan, Hubei, China), anti-Cleaved caspase-9 (1:2000, Abcam, Wuhan, Hubei, China), anti-intercellular adhesion molecule 1 (ICAM-1; 1:5000, Abcam, Wuhan, Hubei, China), anti-vascular cell adhesion molecule-1 (VCAM-1; 1:2000, Abcam, Wuhan, Hubei, China), anti-SCAI (1:1000, Abcam, Wuhan, Hubei, China). After 4°C incubation overnight, the membranes were incubated with the secondary horseradish peroxidase (HRP) antibodies (Sigma–Aldrich) for 1 h. The protein bands were visualized by using the Image Lab (Bio-Rad Laboratories, CA, U.S.A.).

### Oligonucleotide transfection

When cells reached 70% confluence, they were transfected with shRNA, and inhibitors by using the Lipofectamine Transfection Kit (Invitrogen, U.S.A.) following the manufacturer’s instructions. In brief, for silenced expression of miR-317b-5p, inhibitor against miR-317b-5p and NC inhibitor were obtained from Invitrogen (New York, U.S.A.) and have been used. A control shRNA against SCAI gene, as well as control shRNA (sh-NC) were synthesized by Genechem (Shanghai, China) and have been used. Then they were transfected with green fluorescent protein (GFP)-tagged LC3 plasmid into NSCLC cell lines by Lipofectamine 2000 at a final concentration of 100 nM. The expression of GFP was visualized with the scanning fluorescence microscope (Olympus FV1000, Tokyo, Japan).

### CCK8 assay

To determine the cell proliferation, the CCK8 assays were carried out in the present study. After oligonucleotide transfection, cells were seeded into a 96-well plate at the density of 1 × 10^3^ per well and incubated at 37°C for 24, 48 and 72 h. The 10-μl CCK8 solution was added and the cell proliferation was evaluated. The number of proliferative cells was measured by the OD value at 450 nm absorbance by using the microplate reader.

### Wound scratch assay

In the present study, the metastatic ability of the NSCLC cells was determined by the wound scratch assay. First, the transfected cells were seeded into a six-well plate at the density of 1 × 10^5^ per well and incubated at 37°C for 24 h. Then, a linear scratch wound was scraped on the cell monolayer by a pipette tip. The migrated cells and wound healing images were visualized at 24 and 48 h by using the image analysis and detection system.

### Transwell chamber assay

For invasion assay, a total of 1 × 10^5^ cells/well NSCLC cells were seeded in the transwell migration chambers containing a 8-μm-sized porous membrane (Corning, New York, U.S.A.). The upper chamber was inserted with matrigel while the lower chamber was added with 20% FBS. After 48-h incubation at 37°C, the non-invading cells in the upper chamber were removed by cotton wool. The invaded cells in the upper chamber were stained by 0.1% Crystal Violet solution (Sangon Biotech, Shanghai, China), and counted for three times using a microscope (Olympus, Tokyo, Japan).

### Clone formation assay

The transfected cells (1 × 10^5^) were seeded in a six-well plate first and cultured for 14 days. During this process, the medium was replaced for every 2 days. After fixing with 4% formaldehyde, the clones were stained with Crystal Violet (Sangon Biotech, Shanghai, China) for 3 min to visualize the formation of effective clone. An effective clone was defined as having more than 50 cell numbers. The clone formation rate was calculated as follows: clone formation rate (%) = (number of clones/number of seeded cells) × 100%.

### TUNEL assay

The level of cell apoptosis was evaluated by the TUNEL assay using a TUNEL fluorescein isothiocyanate (FITC) kit (Roche, Indianapolis, U.S.A.). Briefly, cells (1 × 10^5^) were fixed with 4% paraformaldehyde (Sangon Biotech, Shanghai, China) and permeabilized with 0.1% Triton X-100 (Sangon Biotech, Shanghai, China). The apoptotic cells were labeled by the cell death detection kit (Roche, Indianapolis, U.S.A.), and the nuclei was stained by the 4′,6-diamidino-2-phenylindole (Roche, Indianapolis, U.S.A.). The apoptotic cells were counted under an Image-Pro Plus software (Media Cybernetics, Rockville, U.S.A.).

### Dual-luciferase reporter gene assay

At the beginning, the potential target of miR-317b-5p was predicated by the TargetScan platform (www.targetscan.org), and the SCAI was detected as one of the possible target of miR-317b-5p. The dual-luciferase reporter gene assay was performed to verify the interaction between miR-317b and SCAI. For luciferase reporter assay, the NSCLC cells were transfected with mutant- or wild-type reporter plasmid containing 3′UTR of SCAI to form the reporter vector SCAI Wt type or SCAT Mut type. Then cells were co-transfected with SCAI Wt or SCAT Mut and miR-371b-5p mimic or NC mimic by using Lipofectamine 2000 reagent (Promega, Madison, U.S.A.). After 48-h culturing, the luciferase activities were detected by the dual-luciferase reporter assay system.

### Statistical analysis

The data were presented as mean ± SD. Statistical analysis was performed by SPSS20.0 software. The overall survival between the different groups of the patients was evaluated by Kaplan–Meier analysis and compared by the log-rank test. The Student’s t-test or one-way analysis of variance (ANOVA) was used to analyze the significance between different groups. *P*<0.05 was considered as statistically significant.

## Results

### MiR-317b-5p is highly expressed in NSCLC tissues and cells

**Figure 1:**
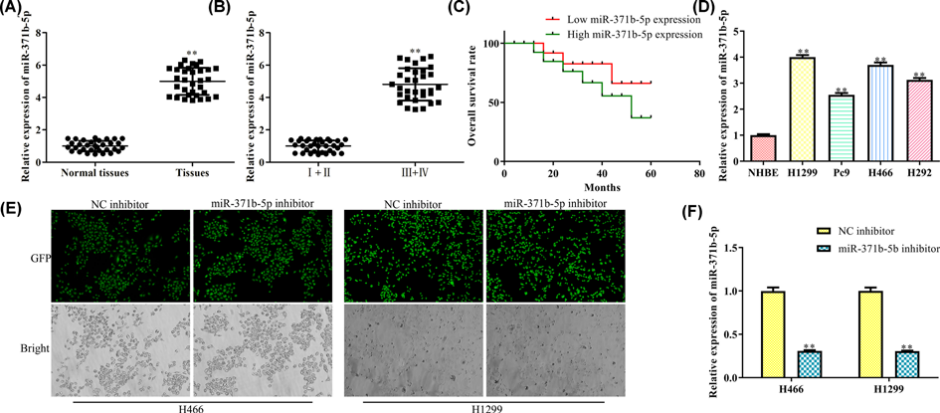
miR-317b-5p is highly expressed in NSCLC tissues and cells (**A**) Relative expression levels of miR-317b-5p in normal NSCLC tissues (*n*=32) was detected by qRT-PCR; **P<0.01 *vs*. Normal tissues. (**B**) Relative expression levels of miR-317b-5p in different tumor stages were detected by qRT-PCR. (**C**) Comparison of overall survival rates between high miR-371b-5p patients and low miR-371b-5p patients (*n*=16). (**D**) Relative expression levels of miR-317b-5p in different cell lines was detected by qRT-PCR; **P<0.01 *vs*. NHBE. (**E**) The transfection efficacy was detected by GFP. (**F**) Relative expression levels of miR-317b-5p in different transfected cell groups. **P<0.01 *vs*. NC inhibitor.

The expression of miR-317b-5p in NSCLC tissues and paired normal tissues were detected by qRT-PCR. As shown in Figure 1A, the expression of miR-317b-5p level was significantly up-regulated in NSCLC tissues compared with the normal tissues (*P*<0.01). The miR-317b-5p level was also shown to be associated with the TNM stages, because high expression level was found in stages III and IV compared with stages I and II (*P*<0.01, Figure 1B). Kaplan–Meier survival curves showed that patients with higher miR-317b-5p had significantly shorter overall survival rate than patients with low expression (*P*<0.01, Figure 1C). Next, we measured the expression of miR-317b-5p in NHBE and human NSCLC cell lines, and found that it was up-regulated significantly in all the human NSCLC cell lines, especially in H1299 and H466 (Figure 1D) in comparison with NHBE. Therefore, we chose these two cell lines (H1299 and H466) for further studies.

To further dissect the effect of miR-371b-5p on the H466 and H1299 cells, the inhibitor of miR-371b-5p was transfected into these cells and the transfection efficiency was detected by GFP expression. As a result, the GFP was expressed in all the transfected cells, that transfected with NC inhibitor or miR-371b-5p inhibitor in both the cell groups (Figure 1E), indicating that a successful transfection model was established. As expected, qRT-PCR showed that the miR-371b-5p was significantly down-regulated in the miR-371b-5p inhibitor-transfected H466 and H1299 cells (Figure 1F).

### Down-regulation of miR-371b-5p inhibits proliferation and promotes apoptosis of NSCLC cells

**Figure 2:**
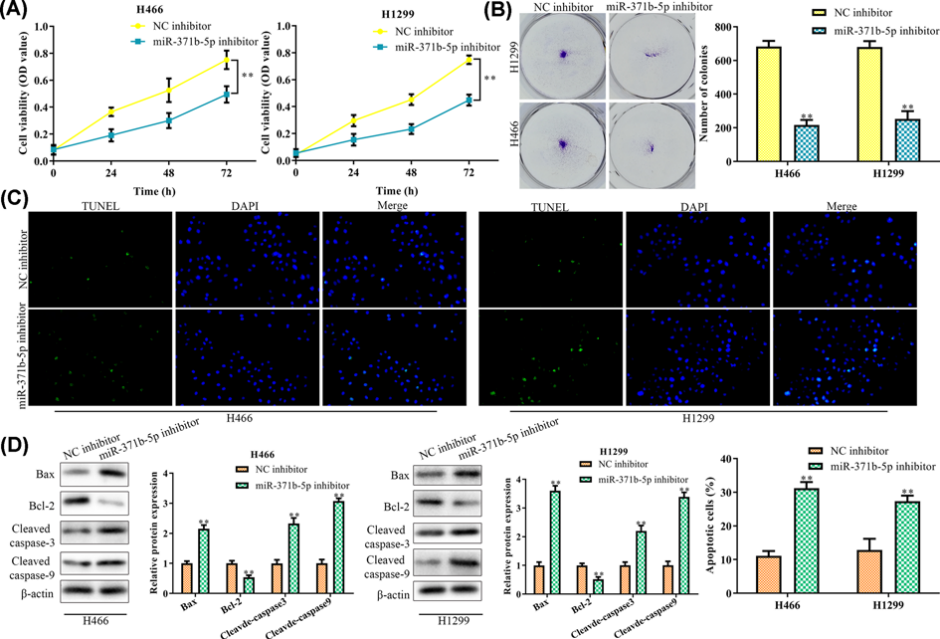
Down-regulation of miR-371b-5p inhibits proliferation and promotes apoptosis of NSCLS cells (**A**) Cell viability was detected by the CCK8 assay. (**B**) Number of clones was detected by clone formation assay. (**C**) Cell apoptosis was detected by the TUNEL assay. (**D**) Expression levels of apoptosis related proteins were detected by the Western blot analysis. **P<0.01 *vs*. NC inhibitor.

To detect the effect of miR-371b-5p inhibitor on cell viability and proliferation, the CCK8 and clone formation assays were conducted. We demonstrated that both the cell viability and the proliferation were significantly reduced when induced by miR-371b-5p inhibitor (Figure 2A,B). TUNEL assay showed that the miR-371b-5p inhibitor promoted cell apoptosis in both the NSCLC cell lines (Figure 2C). We then detected the apoptosis-related proteins and found that the proteins Bax, Cleaved caspase-3 and Cleaved caspase-9 were all up-regulated in miR-371b-5p inhibitor-transfected cell group, while the anti-apoptotic protein Bcl-2 was down-regulated in this group (Figure 2D), indicating that miR-371b-5p inhibitor induced cell apoptosis by activation of apoptosis-related proteins.

### MiR-371b-5p inhibitor inhibits migration and invasion of NSCLC cells

**Figure 3:**
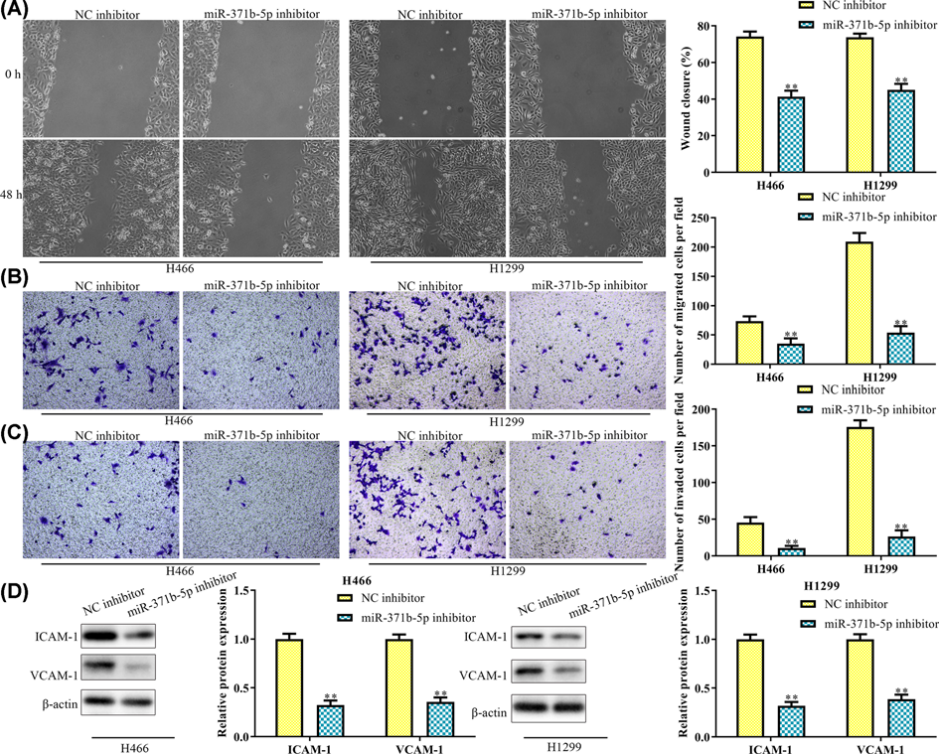
miR-371b-5p inhibitor inhibits migration and invasion of NSCLC cells (**A**) Wound closure rate was detected by the scratch wound healing assay. (**B**) Cell migration was detected by the transwell assay. (**C**) Cell invasion was detected by the transwell assay. (**D**) Expression levels of ICAM-1 and VCAM-1 were detected by the Western blot analysis. **P<0.01 *vs*. NC inhibitor.

Since interfering miR-371b-5p in H466 and H1299 cells suppressed the invasion and proliferation of NSCLC cells, we then interfered miR-371b-5p *in vivo* to detect the effect on the migration and invasion in H466 and H1299 cells. From the scratch wound healing assay and transwell assay, we found that the number of invasive cells and migratory cells in the miR-371b-5p inhibitor group was significantly decreased compared with that of the NC inhibitor group (Figure 3A–C). As a group of adhesion molecules, the ICAM-1 and VCAM-1 were detected in the present study to verify the effect of miR-371b-5p inhibitor on migration and invasion of NSCLC cells. Western blot analysis showed that both these adhesion molecules were down-regulated in the miR-371b-5p inhibitor group in H466 and H1299 cells (Figure 3D). All the above results suggested that the miR-371b-5p inhibitor inhibited migration and invasion of NSCLC cells.

### MiR-371b-5p targets SCAI and causes post-transcriptional suppression

**Figure 4:**
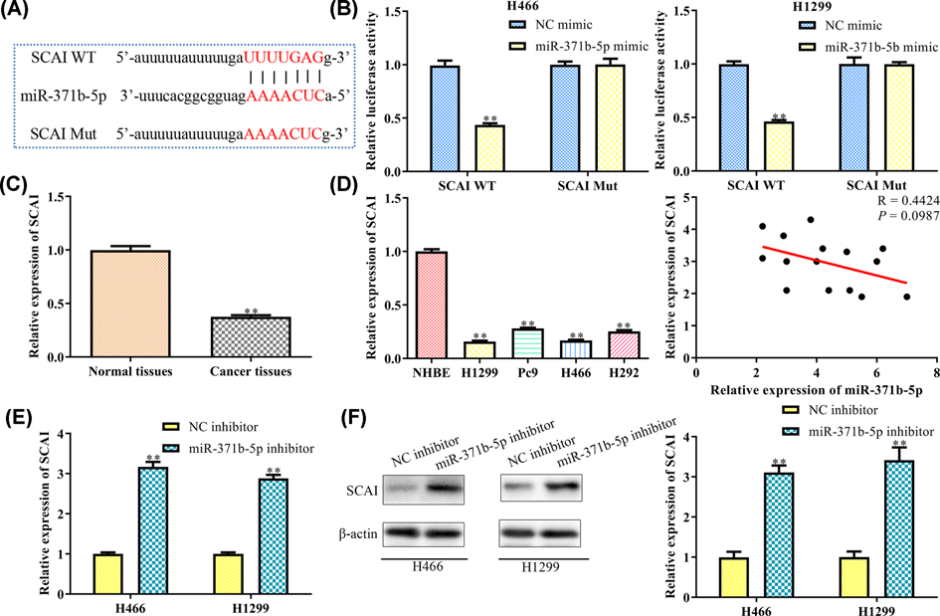
miR-371b-5p targets SCAI and causes post-transcriptional suppression (**A**) Binding site of miR-371b-5p was analyzed by the TargetScan platform. (**B**) Relative luciferase activity was detected by dual-luciferase reporter assay. **P<0.01 *vs*. NC mimic. (**C**) Relative expression of SCAI in NSCLC tissues and cell lines were detected by Western blot analysis. **P<0.01 *vs*. Normal tissues. (**D**) Relationship between relative expression of SCAI and miR-371b-5p. **P<0.01 *vs*. NHBE. (**E**) Relative expression of SCAI in different transcription groups was detected by qRT-PCR. (**F**) Relative expression of SCAI in different transcription groups was detected by Western blot analysis. **P<0.01 *vs*. NC inhibitor.

In the preliminary experiment, we identified that the SCAI is one of the potential target genes by TargetScan platform. As shown in Figure 4A, the 3′UTR of the SCAI has one binding site of miR-371b-5p. The regulatory effect of miR-371b-5p and SCAI was further validated by the luciferase assay. Results showed that miR-371b-5p significantly attenuated the luciferase reporter activity of the SCAI 3′ UTR other than the SCAI Mut sequence (Figure 4B). These results confirmed that the SCAI was a direct target of miR-371b-5p in H466 and H1299 cells. We then detected the expression level of SCAI mRNA in the NSCLC tissues and H466 and H1299 cell lines (Figure 4C), and found that SCAI was decreased in cancer cells than in the normal cells. Correlation analysis demonstrated a negative correlation between miR-371b-5p and SCAI expression (*P*<0.05, Figure 4D). The expression level of SCAI was increased apparently at transcriptional and translational protein levels in both H466 and H1299 cell lines transfected with miR-371b-5p inhibitor (Figure 4E,F).

### Down-regulation of SCAI rescues cell proliferation and inhibits cell migration and invasion caused by miR-371b-5p inhibitor

**Figure 5:**
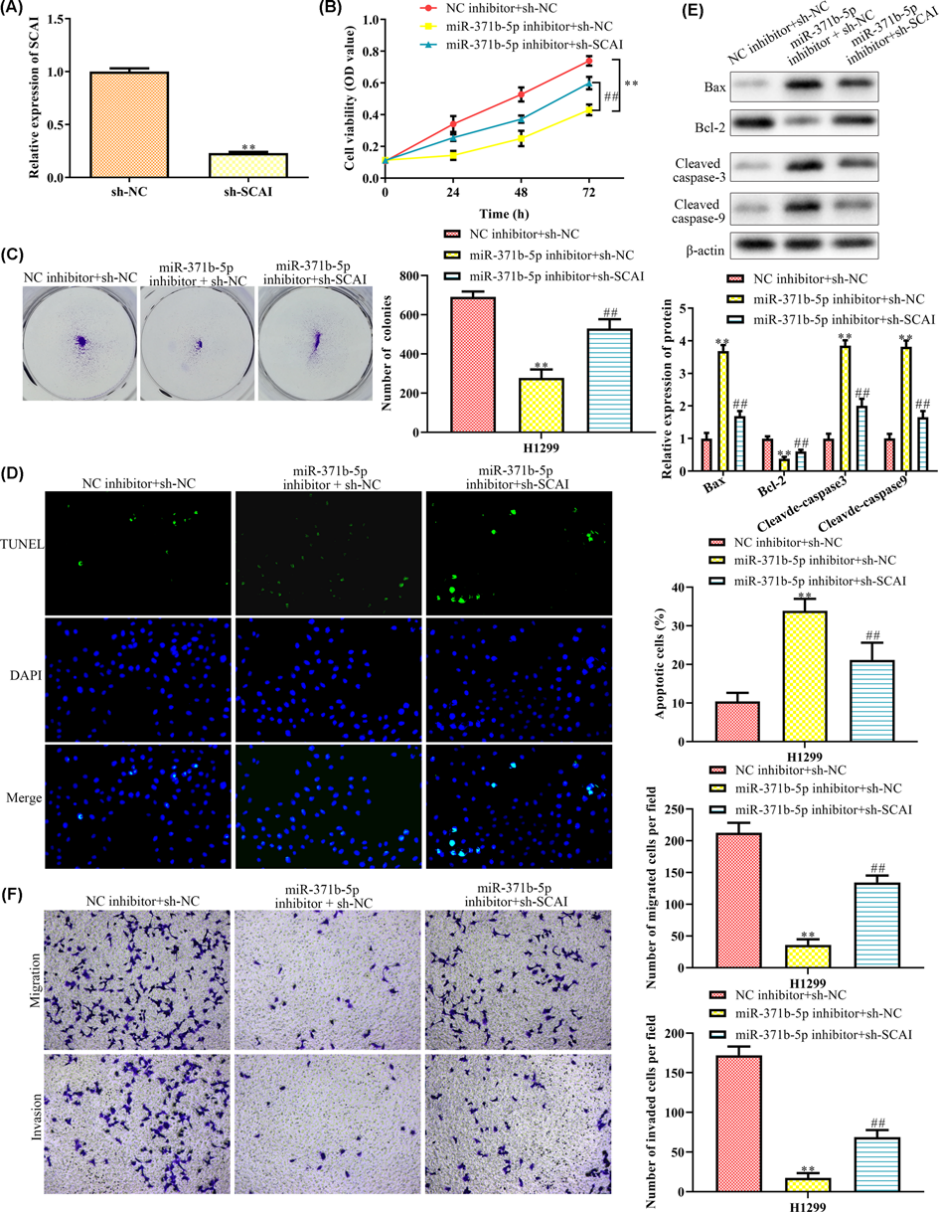
Down-regulation of SCAI rescues cell proliferation and inhibits cell migration and invasion caused by miR-371b-5p inhibitor (**A**) Relative expression of SCAI in different transcription groups nwas detected by qRT-PCR. (**B**) Cell viability was detected by the CCK8 assay. (**C**) Number of clones was detected by clone formation assay. (**D**) Cell apoptosis was detected by the TUNEL assay. (**E**) Relative expressions of apoptosis-related proteins were detected by Western blot analysis. (**F**) Cell migration and invasion were detected by transwell chamber assay. **P<0.01 *vs*. NC inhibitor + sh-NC. ^##^*P*<0.01 *vs*. NC inhibitor + sh-NC.

To further verify if the SCAI was associated with the cell proliferation, migration and invasion via modulating miR-371b-5p, we transfected sh-SCAI into H1299 cells treated with miR-371b-5p inhibitor. First, the relative expression of SCAI was compared between the sh-NC group and sh-SCAI group to detect if the SCAI interference is successful. As a result, the SCAI expression was significantly down-regulated in sh-SCAI group (Figure 5A), indicating a successful interference is established. From the results of the CCK8 assay (Figure 5B) and clone formation assay (Figure 5C), we found that the sh-SCAI suppressed the decrease in cell viability and cell proliferation caused by miR-371b-5p inhibitor. Similarly, the interference of SCAI also inhibited the number of apoptotic cells induced by miR-371b-5p inhibitor (Figure 5D). The effect on cell apoptosis was verified by the expression of apoptosis related proteins, that the apoptosis-related proteins (Bax, Cleaved caspase-3 and Cleaved caspase-9) were significantly down-regulated while the anti-apoptosis protein Bcl-2 was significantly up-regulated in the cells co-treated with miR-371b-5p inhibitor and sh-SCAI (Figure 5E). Transwell chamber assay showed that although the migration and invasion of the H1299 cells were inhibited by miR-371b-5p inhibitor, this alteration was reversed by knockdown of SCAI (Figure 5F). These results indicated that the down-regulation of SCAI inhibited cell migration and invasion caused by miR-371b-5p inhibitor.

## Discussion

The miRNAs has been demonstrated to play critical role either as tumor suppressor or as oncogene in regulation of human NSCLC cells [18–20]. In the current study, we evaluate the expression levels of miR-371b-5p in human NSCLC, and discovered that the miR-371b-5p was aberrantly up-regulated in both human NSCLC tissues and NSCLC cell lines. This result is in assistance with the previous data that the miR-371b-5p was up-regulated in human cancers [16,17]. We also found that the silencing of this miRNA could significantly inhibited cell proliferation and viability, migration and invasion in H1299 and H466 cell lines via directly targeting SCAI. Therefore, we concluded from our results that the miR-371b-5p is regarded as a potential biomarker and therapeutic target in treatment of NSCLC.

In the present study, we discovered that the down-regulation of miR-371b-5p inhibited cell proliferation, and promoted cell apoptosis, suppressed migration and invasion in NSCLC cells. To understand the molecular mechanism of miR-371b-5p on cell apoptosis, we detected the expression of apoptosis-related genes (Bax, Bcl-2, Cleaved caspase-3 and Cleaved caspase-9) in both H466 and H1299 by Western blot. Results showed that the expression levels of pro-apoptotic factor (Bax), Cleaved caspase-3 and Cleaved caspase-9 were all significantly up-regulated while the expression of anti-apoptotic factor (Bcl-2) was significantly down-regulated in NSCLC cells when treated by miR-371b-5p inhibitor. These results indicated that the miR-371b-5p inhibitor induced cell apoptosis by activating the Cleaved caspases-3/8-related pathway.

Metastasis is induced by abnormal expressions of proto-oncogene and tumor suppressor gene, which will result in cancer cell proliferation, migration, and invasion [21,22]. Therefore, it is one of the major causes of cancer related mortality. In order to understand the molecular mechanism of miR-371b-5p on metastasis, we discovered the expression level of adhesion molecules (ICAM-1 and VCAM-1). As a result, both of them were significantly down-regulated in the miR-371b-5p interfered cell group, suggesting that the down-regulation of miR-371b-5p inhibited cell adhesion ability thus resulted in reduced cell migration and invasion. Expression of VCAM-1 and ICAM-1 seems to be closely related to the metastasis of cancer cells. For instance, Min and colleagues [23] found that VCAM-1 was overexpressed in metastatic breast cancer cells to lungs. It also been identified to be up-regulated in colorectal cancer, and is correlated with the tumor progression and lymph node metastasis [24]. ICAM-1 was targeted by miR-147 and thereby affected the cell migration of gastric cancer cells [25]. Therefore, we concluded from our results that the miR-371b-5p inhibitor inhibited cell migration and invasion by regulation of ICAM-1 and VCAM-1 expression in NSCLC cells.

The human miR-371b-5p is located at 19q13.42 and belongs to the member of miR-371b family. It plays a key role in many human diseases. In order to identify its target gene during these biological processes, we performed the bioinformatics analysis. We first predicated the SCAI as one of the promising target gene of miR-371b-5p via TargetScan platform, then we performed the dual-luciferase report assay to validate the predication, and found that the activity of luciferase reporter fused with Wt SCAI 3′UTR, but not fused with Mut SCAI 3′ UTR. By using qRT-PCR, we discovered that the expression of SCAI was down-regulated in NSCLC cancer cells, and negatively associated with the expression of miR-371b-5p.

SCAI is located at the 9q33.3 position and can act as a suppressor during cancer cell metastasis. It is verified to be down-regulated in glioma which then promotes cell migration by stimulating the Wnt/β-catenin pathway [26]. In oral squamous cell carcinoma cells, SCAI is targeted by miRNA-25-3p and induces cell migration by activating the E-cadherin pathway [27]. In papillary thyroid carcinoma cells, SCAI mediated cell proliferation and migration by binding to miR-574-5p via Wnt/β signaling pathway [28]. Our results also presented a low expression of SCAI in NSCLC cell lines, indicating that this gene could be used as a diagnostic factor for NSCLC patients.

In summary, the present study revealed that the miR-371b-5p is significantly up-regulated in NSCLC tissues and cells, and is correlated with the down-regulation of SCAI expression. The protective effect of miR-371b-5p inhibitor against NSCLC cell proliferation, migration and invasion is mediated via binding to SCAI. Therefore, the miR-371b-5p and SCAI may serve as biomarkers or potential targets for novel therapeutic targets for treatment of NSCLC.

## Data Availability

The raw/processed data required to reproduce these findings cannot be shared at this time as the data also form part of an ongoing study.

## Abbreviations

ATIIC: alveolar progenitor type II cell
Bax: BCL2 associated X
Bcl-2: B cell leukemia/lymphoma 2
CCK8: Cell Counting Kit-8
FBS: fetal bovine serum
FUT4: fucosyltransferase 4
GFP: green fluorescent protein
HMGA2: high mobility group AT-hook 2
ICAM-1: intercellular adhesion molecule 1
MAP3K11: mitogen-activated protein kinase kinase kinase 11
miRNA: microRNA
NHBE: normal human bronchial epithelial cell
NSCLC: non-small cell lung cancer
OD: optical density
qRT-PCR: quantitative reverse transcription-polymerase chain reaction
SCAI: suppressor of cancer cell invasion
TNM: tumor node metastasis
TUNEL: terminal dexynucleotidyl transferase(TdT)-mediated dUTP nick end labeling
VCAM-1: vascular cell adhesion molecule-1
3′UTR: 3′ untranslated region
